# Evaluation of High-Frequency Measurement Errors from Turned Surface Topography Data Using Machine Learning Methods

**DOI:** 10.3390/ma17071456

**Published:** 2024-03-22

**Authors:** Przemysław Podulka, Monika Kulisz, Katarzyna Antosz

**Affiliations:** 1 Faculty of Mechanical Engineering and Aeronautics, Rzeszow University of Technology, 35-959 Rzeszow, Poland; p.podulka@prz.edu.pl; 2 Faculty of Management, Lublin University of Technology, 20-618 Lublin, Poland; m.kulisz@pollub.pl

**Keywords:** surface topography, roughness, machining, turning, high-frequency errors, measurement noise, artificial neural network, SVM, decision trees

## Abstract

Manufacturing processes in industry applications are often controlled by the evaluation of surface topography. Topography, in its overall performance, includes form, waviness, and roughness. Methods of measurement of surface roughness can be roughly divided into tactile and contactless techniques. The latter ones are much faster but sensitive to external disturbances from the environment. One type of external source error, while the measurement of surface topography occurs, is a high-frequency noise. This noise originates from the vibration of the measuring system. In this study, the methods for reducing high-frequency errors from the results of contactless roughness measurements of turned surfaces were supported by machine learning methods. This research delves into optimizing filtration methods for surface topography measurements through the application of machine learning models, focusing on enhancing the accuracy of surface roughness assessments. By examining turned surfaces under specific machining conditions and employing a variety of digital filters, the study identifies the Gaussian regression filter and spline filter as the most effective methods at a 22.5 µm cut-off. Utilizing neural networks, support vector machines, and decision trees, the research demonstrates the superior performance of SVMs, achieving remarkable accuracy and sensitivity in predicting optimal filtration methods.

## 1. Introduction

When analysing surface topography, significant amount of information can be obtained about the robustness of the manufacturing process using in-process controls [1], the prediction of accuracy of roughness generation in ultra-precision machining [2], as a quality control tool [3], or, generally, the quality of the surface indicator [4]. Topography can also be suitable for the study of entire bending-fatigued fractured surfaces of specimens obtained by explosive welding [5]. In practice, the roughness can be studied for any case where the functional properties of a surface are considered [6].

Roughly, the techniques of surface topography measurement can be divided into tactile and contactless. Historically, diamond contact stylus methods were found suitable when studying dental tissue materials [7]. The selection criteria for the stylus techniques, including a tip radius to improve the reliability of measurement results was comprehensively studied considering 2D and 3D distortion effects [8]. Tribologically, profilometric measurement of wear scars [9] or low wear [10] allowed a better understanding of the rough surface performances. The non-contact measuring techniques have become extremely popular due to their reduction in time [11]. Many studies of scholarly and industrial centres are based on contactless systems when measuring surface topography [12]. Their advantages over tactile techniques were reviewed and highlighted in many application comparisons [13,14,15].

Despite the many benefits of contactless measurement, these methods are fraught with many errors, affecting the accuracy of the whole measuring process. A significant type of measurement error is measurement noise [16]. From the definition, measurement noise is an error added to the output signal when the normal use of a measuring instrument occurs [17]. Roughness measurement noise can be characterised along with its bandwidth [18], finding one of the often-detected errors in the high-frequency domain. The main source of high-frequency noise presence is an environment-induced vibration [19,20]. Selected ISO 25178 roughness parameters are most susceptible to high-frequency noise occurrence [21], but the comprehensive characterisation of surface properties with a basis of the topography evaluation cannot be achieved without analysing the measured accuracy.

The vibration was effectively studied with the application of an artificial neural network (ANN) with the simulation of selected roughness parameters [22]. The statistical characterisation was found to be suitable for milled magnesium alloy roughness parameters supported by ANN methods [23]. ANN techniques are encouraged for studying surface topography as they use a simple optical device [24]. Furthermore, milled surface roughness profiles can be predicted with a connection of ANN and fractal geometry [25]. Generally, the machined surfaces can be thoroughly examined or modelled using an ANN [26], considering modern data analysis techniques.

The study of turned surfaces was found significant in many industrial applications, including aviation [27]. In terms of grinding, turned surfaces are often applied in many automotive part manufacturing processes [28]. Roughness and geometric tolerance were defined as crucial in the comparison of dry and wet turning contrary to the minimum quantity lubricant (MQL) machining [29]. Monitoring of tool conditions while machining [30] requires more comprehensive studies and machine systems from manufacturers to proceed more flexibly [31]. Turning manufacturing processes for industrial parts were also studied for many composite [32] and ceramic [33] materials when evaluating their roughness performances. Reducing the data processing errors for roughness parameters is especially important when optimizing the cutting conditions [34].

Although many statistical studies have been conducted [35,36,37] and many procedures have been proposed, the guidance on how completely reduce the influence of selected types of topography measurement errors is still unclear. One of the proposed approach is to remove the unwanted data from raw measurements [38].

One of the most challenging tasks to be performed when characterising the raw measured surface topography results is reducing the measurement noise [39]. Even if a highly precise measuring device is applied [40], the relevance of the final result acquisition can be lost when data are processed and described inaccurately [41]. Since the detailed and sophisticated study of measurement noise is time-consuming [42], the paper deals with the application of machine learning methods for the selection of proper methods for error reduction in surface topography measurements.

Thus, the main goal of this study was to optimise the selection of the filtration method and the cut-off size to maximise the reduction in measurement/image distortion. The novelty of these studies is the use of a classifier that, based on measurement data, i.e., roughness measurement, determines the appropriate filtering method and cut-off size to achieve the highest reduction in measurement/image distortion. For this purpose, in this study, the machine learning methods such as neural network (NN), support vector machine (SVM), and decision trees (DT) were used to select the best solution for noise filtering. The article begins with an introduction, continues with a section outlining the research methods and materials (Section 2), and then presents the results of the developed machine learning models (Section 3). It concludes with conclusions (Section 4), limitations, and future research (Section 5).

## 2. Materials and Methods

### 2.1. Analysed Surfaces

**Figure 1:**
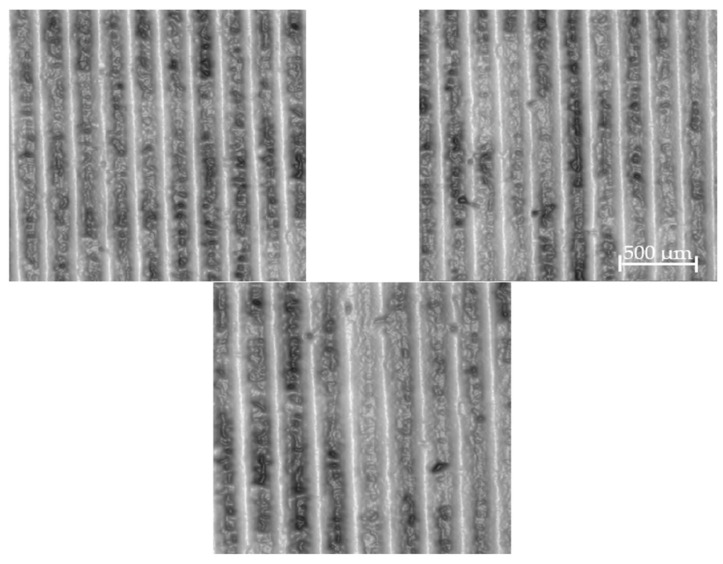
Examples of topography images of turned surfaces with a machining cross-hatch angle approximately equal to 85°.

The turned surfaces made with cast iron were measured and studied. In the machining, the cutting speed was 50 m/min, the feed rate was 0.1 mm/rev, and the depth of cut was 0.25 mm. For the cutting tool geometries, the corner (edge) radius was 0.8 mm, and the rake angle was 6°. More than 20 surfaces were studied, and examples were presented in detail. In Figure 1, the examples of images of analysed turned surfaces are provided.

The cast iron material was recognised as promising and straightforward with optimisation when the cutting conditions in hard turning were investigated [43]. Various cast iron materials were compared and encouraged for use when studying flank wear dry conditions during turning processes [44]. The tool wear when turning grey cast iron using carbide cutting inserts was improved with machine learning methods, implying the prediction in the tool conditions [45]. Historically, when machining cast iron materials, the ceramic tool performance can address most of the wear resistance performance [46]. Except for recyclability, low lifecycle energy consumption, and low costs, cast iron has a good wear resistance, which is often applied in many turned surfaces with tribological designation [47]. The high abrasion resistance was also classified as sufficiently profitable including erosive environments [48]. Therefore, based on their historical significance, cast iron materials are involved in many industrial and engineering applications [49].

### 2.2. Measurement Process

All the studied details were measured with the contactless instrument, the white light interferometer (WLI), Talysurf CCI Lite, produced by Taylor Hobson Ltd., Leicester, UK, version 2.8.2.9. It was employed with the following parameters/features: a height resolution of 0.01 nm, a received area of 3.35 by 3.35 mm^2^ with 1024 × 1024 measured points, respectively, a spacing of 3.27 μm, and a Nikon 5×/0.13 TI objective.

For the analyses of the roughness, the following areal digital filters from the TalyMap Gold version 6.1 software, copyright by Digital Surf, were employed to obtain the ISO 25178 texture parameters: Gaussian regression filter (GRF), robust Gaussian regression filter (RGRF), and isotropic spline filter (SF). The digital filter based on the Gaussian function was introduced in 1996, with the ISO 11562 standard [50]. From this period onwards, it became one of the most often-used approaches for the characterisation of surface roughness [51]. Accompanying the morphological filtering techniques, the Gaussian regression filter was used for the separation of form, waviness, and roughness from the powder bed fusion (PBF) in a recently popular additive manufacturing (AM) method [52]. In addition to the regular Gaussian filtering techniques, the robust modification of the weighting function was defined and modified in the further editions of ISO 16610-31 [53]. Compared to the regular Gaussian and spline filters, it was found to be more accurate when analysing engineering surface topography by applying an extended discrete modal decomposition approach [54]. The isotropic spline filter was found to more advanced than that used in the traditional Gaussian methods and was incorporated into the ISO standard as a substitute [55].

**Figure 2:**
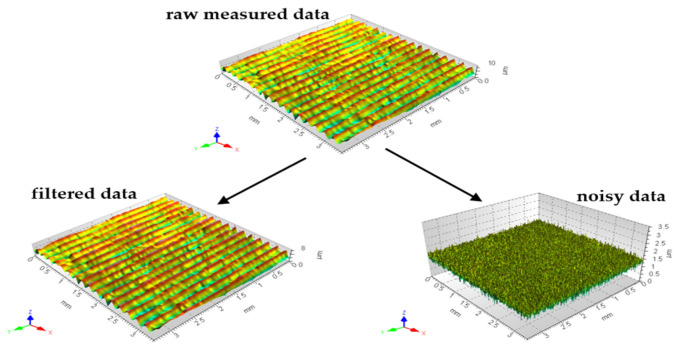
Examples of surface topography data decomposition by application of GRF with cut-off of 15 µm.

Thus, all the applied data filtering algorithms were received and validated by using this source. In Figure 2, the examples of data decomposition are presented with the application of the GRF.

### 2.3. Machine Learning Methods

To develop the prediction models, three different machine learning methods were used: neural network (NN), support vector machine (SVM), and decision trees (DT).

***Neural networks (NNs)*** stand as a prominent category within the realm of machine learning techniques, extensively utilised for tasks such as classification and regression analysis. The architecture of a neural network is intricate, comprising various components including input nodes, weight parameters, aggregation functions, activation functions, and output elements. In the context of multilayer networks, the process begins with the input layer receiving data from the training dataset’s predictors. This is followed by one or more hidden layers, where each neuron’s input is the output from the previous layer, transformed by activation functions. These functions are essential for introducing non-linearity into the network, allowing it to model complex relationships [56].

The aggregation function, often a linear combination of inputs and their corresponding weights, plays a critical role in the network’s operation. It determines the weighted sum of the inputs, which is then directed through an activation function to produce the neuron’s output. This output can either be passed to the next layer in the network or, in the case of the final layer, serve as the model’s prediction output [57,58].

The aggregation function computes the weighted sum of inputs and their corresponding weights for each neuron in a layer. For a given neuron *j* in layer *l*, this can be represented as (1):

(1) $$z_{j}^{\left(l\right)}=\sum_{i}w_{ij}^{\left(l\right)}x_{i}+b_{j}^{\left(l\right)}$$

where $z_{j}^{\left(l\right)}$ is the aggregated input to neuron *j* in layer *l*, $w_{ij}^{\left(l\right)}$ represents the weight from neuron *i* in the previous layer to neuron *j* in the current layer, *x_i_* is the input from neuron *i* or the input feature if *l* is the input layer, and $b_{j}^{\left(l\right)}$ is the bias term for neuron *j* in layer *l*.

The activation function introduces non-linearity to the model, allowing it to learn complex patterns. The output of neuron *j* in layer *l*, after applying the activation function *f*, is (2):

(2) $$a_{j}^{\left(l\right)}=f(z_{j}^{\left(l\right)})$$

Common activation functions include ReLU (rectified linear unit), sigmoid, and tanh, each with different mathematical expressions, e.g., *f*(*z*) = max (0, *z*) for ReLU.

Neural networks are distinguished by their ability to learn and model non-linear and complex relationships, making them highly effective for a wide range of applications, from image and speech recognition to forecasting and beyond. The learning process involves adjusting the weights of connections based on the error between the predicted and actual outputs to minimise the prediction error across the training data. This adaptability and depth of learning capability underline the neural network’s significance in advancing machine learning and artificial intelligence fields.

***Support Vector Machine (SVM)*** is an advanced machine learning model widely used in classification and regression, especially in scenarios where the feature space is large, and the data can be separated linearly or non-linearly. The primary goal of SVM is to find the hyperplane that best separates data belonging to different classes. This separation is based on maximising the margin, which is the distance between the hyperplane and the closest points from each class, known as support vectors. In cases where the data are not linearly separable, SVM uses kernel functions such as linear, polynomial, radial basis function (RBF), or sigmoid to transform the data into a higher dimensional space where it can be linearly separated [58,59].

A key feature of SVM is its ability of solving the optimisation problem, which involves finding model parameters that minimise the cost function while maximising the margin and imposing penalties for classification errors. This method is valued for its effectiveness in solving complex classification tasks, especially with high-dimensional data. However, it also requires relatively high computational effort, especially for large datasets, and precise selection of the kernel function and its parameters.

In linear classification, the main goal of SVM is to find a separating hyperplane, defined by the equation: *w* ∗ *x* + *b* = 0, where *w* represents the weight vector, *x* is the feature vector, and *b* is the bias term. SVM aims to maximise the margin, the distance between the hyperplane and the nearest data points from both classes, known as support vectors, leading to two equations for the support vectors: *w* ∗ *x*_+_ + *b* = 1 for one class and *w* ∗ *x*_−_ + *b* = 1 for the other [59].

The optimisation problem in SVM involves minimizing the expression (3):

(3) $$\frac{1}{2}{\left||x|\right|}^{2}+C\sum_{i=1}^{n}\xi i$$

where *C* is a regularisation parameter controlling the trade-off between maximizing the margin and minimizing classification error, and *ξi* are slack variables corresponding to classification errors for individual data points.

SVMs are used in various fields, including pattern recognition, image analysis, text classification, and bioinformatics, due to their ability to efficiently classify and regress in complex feature spaces. Despite a few challenges in their application, support vector machines remain one of the most powerful tools in machine learning, offering a unique combination of precision and adaptability.

***Decision trees (DTs)*** are a data-modelling technique applicable to both classification and regression tasks. This approach facilitates analysis to derive logical “if, then” rule conditions that help to accurately classify the entities being analysed. In data mining and machine learning, decision trees act as predictive models and are among the most popular and efficient data-mining techniques, often used to make predictions. When the dependent variable is qualitative, classification trees are generated, whereas regression trees are developed for cases where the dependent variable is continuous. Classification trees aim to classify objects into specific classes, relying on one or more explanatory variables to assess their influence on a qualitative dependent variable—essentially the variable to be predicted. The prediction is conceptualised as a model capable of estimating the value (or range of values) of a characteristic, which may include, in particular, a class label. The decision tree construction process involves an exhaustive exploration of all variables and potential divisions within a dataset for each decision node (*t*), intending to identify the most advantageous division [60,61].

The authors of the algorithm recommend employing the Gini index, also known as the measure of node impurity or pollution. They propose partitioning the entire k-dimensional space, *R^k^*, into *q* distinct regions, such that $R_{1}\cup R_{2}\cup \ldots \cup R_{q}=R^{k}$. For a given node *m*, where 1≤m≤q, corresponding to the region *R_m_*, the Gini index is calculated in the following manner (4):

(4) $$Q_{G}(m)=\sum_{j=1}^{s}p_{mi}(1-p_{mi})=1-\sum_{j=1}^{s}p_{mi}^{2}$$

where *p_mi_* denotes the conditional probability of the *j*-th class within a node, and *s* represents the total number of classes. For node *m*, which contains *n_m_* observations, the conditional probability for the *j*-th class is given by (4):

(5) $$p_{mi}=\frac{\#\{y=c_{i}:x\in R_{m}\}}{n_{m}}$$

### 2.4. Modelling Methodology

The main goal of the research was to optimise the selection of the filtration method and the cut-off size. Therefore, it was proposed to train a classifier that, based on the measurement data, i.e., roughness measurement, determines the appropriate filtering method to be used. In particular, the classifier suggests which filtering method and cut-off size should be used to obtain the highest reduction in measurement/image distortion. The first phase of the research consisted of preparing test samples using different filtering methods: GRF—Gaussian regression filter; RGRF—robust Gaussian regression filter; SF—spline filter; and FFTF—fast Fourier transform filter. Different cut-off values were analysed for each of the aforementioned filtration methods: 2.5 µm, 5 µm, 7.5 µm, 10 µm, 12.5 µm, 15 µm, 17.5 µm, 20 µm, 22.5 µm, and 25 µm. The second stage of the research was to generate training data to train the classifier. Data were generated using the following procedure:

1. For each case in the dataset, using the quality indicators presented in Table 1, the filtration method and cut-off value for which the quality of the obtained image is the best were selected.
2. Then, the index of the optimal model for each case was written in the designated table. The assignment of models to indexes is presented in Table 2.

The selection of quality indicators such as mean square error (MSE), peak signal-to-noise ratio (PSNR), structural similarity index (SSIM), and image correlation coefficient (ICC) in research that focused on optimizing the filtration method and cut-off size provides a comprehensive assessment of image reconstruction quality. MSE is used to evaluate the average squared error between the original and reconstructed images, offering a straightforward measure of the total error introduced by filtration. PSNR assesses the quality of reconstruction in the context of noise levels, where higher values indicate better quality. SSIM, considering perceptual aspects such as contrast and structure, allows for the evaluation of the visual quality of the image, aligning with human perception of quality. ICC measures the degree of statistical correlation between the original and reconstructed images, crucial for assessing the preservation of patterns and structures. Together, these indicators offer a holistic view of the impact of filtration on reconstruction quality, enabling the precise determination of the best filtration method and optimal cut-off size.

For MSE, lower values are preferable as they indicate a lower average squared error between the original and reconstructed images, signifying minimal distortion introduced by the filtration process. Conversely, for PSNR, SSIM, and ICC, higher values are sought after. Higher PSNR values suggest a better quality of reconstruction relative to the level of noise, implying that the signal’s fidelity is maintained despite the presence of noise. Similarly, higher SSIM values denote the strong preservation of visual structures in the image, reflecting an alignment with the original image’s perceptual attributes such as texture and contrast. Higher ICC values indicate a high degree of statistical correlation between the original and reconstructed images, ensuring that the filtration method retains the original image’s patterns and structural integrity. The optimal selection involves a balance, wherein MSE is minimised, while PSNR, SSIM, and ICC are maximised, thereby achieving the highest reconstruction quality.

**Figure 3:**
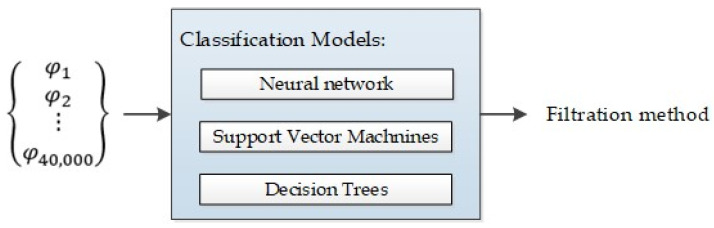
Diagram of the models used for neural networks, SVMs, and decision trees.

The next stage of the research was to train a classifier, based on the measurement data, i.e., roughness measurement, to determine the appropriate filtering method and cut-off size to be used. This classifier is designed to select the appropriate model index for data reconstruction purposes. To train the classifier, a total of 75 datasets were employed. The modelling process was conducted within the Matlab 2023b framework, where an analysis encompassing three distinct machine learning methodologies, i.e., NN, SVM, and DT, was carried out. The diagram of the models used for neural networks, SVMs, and decision trees is shown in Figure 3. The dataset was partitioned into a training subset and a validation subset, adhering to a ratio of 75% to 25%, respectively.

The selection of NN, SVM, and DT for the classifier training process was underpinned by their distinct strengths and capabilities in handling complex pattern recognition and classification tasks. NNs, with their deep and flexible structures, exhibit an exceptional capability to model complex, non-linear relationships between input and output data. This attribute makes them indispensable in adapting to subtle patterns in data, crucial for accurately predicting suitable filtering methods and determining cut-off sizes based on roughness measurements. Their ability to learn from data and continuously improve as more data are accumulated renders NNs an invaluable tool for the task of model index selection for data reconstruction. SVMs, known for their outstanding efficiency in handling classification problems in high-dimensional spaces, excel at identifying the optimal hyperplane that separates different classes. This ability is especially valuable in situations where the relationships between roughness measurements and filtering parameters are complex and not easily separable linearly. The use of SVMs has enabled the precise differentiation of filtering methods in the context of this research. DTs, offering simplicity and high interpretability, allow for easy understanding and visualisation of the decision-making process. Their hierarchical structure facilitates the identification and analysis of the most relevant data features influencing the choice of filtering methods and cut-off sizes. The intuitiveness and visualisation capabilities of DTs are irreplaceable in the initial phase of analysis, helping to clarify which factors are most significant for classification. The incorporation of these three methods into the study was the result of the careful evaluation of their potential applications in the context of surface topography analysis. Other popular machine learning methods were considered, yet the final selection of NNs, SVMs, and DTs was made based on their unique properties that best meet the challenges of this study. This approach was also supported by empirical experiments that confirmed the effectiveness of these methods in the context of the issue at hand, providing a solid basis for the methodological decisions. Future work intends to continue exploring and evaluating various machine learning methods to better understand their applicability in similar research tasks.

In the context of NN modelling, a carefully designed shallow network architecture was employed. This architecture featured a single hidden layer, which is a quintessential element in neural network design and is renowned for its ability to capture complex relationships with significantly lower computational requirements compared to more elaborate networks. The number of neurons within this hidden layer varied between 2 and 20, a decision guided by experimental selection methodology. This approach involved testing different configurations to find the optimal number of neurons that strikes an ideal balance between model complexity and efficiency. Furthermore, the scaled conjugate gradient backpropagation learning algorithm was used to train the network, due to its effectiveness in handling the learning process of the model.

The SVM classifiers underwent a training process utilizing a diverse array of kernel functions, including linear, Gaussian, RBF (radial basis function), and polynomial. Critical parameters, namely, kernel scale, box constraint, and epsilon, were configured to automatic settings, permitting the algorithm to optimise these values for enhanced performance. Furthermore, a parameter optimisation strategy employing cross-search techniques was implemented. To ensure data uniformity in terms of scale and distribution across all SVM models, standardisation procedures were rigorously applied.

The DT models were developed through a methodical construction process, leveraging a variety of splitting criteria, such as Gini impurity, entropy (information gain), and chi-square. Essential parameters, including the maximum depth of the tree, minimum split samples, and minimum leaf samples, were dynamically adjusted, enabling the algorithm to refine these thresholds for optimal model complexity and prevent overfitting. Furthermore, the process involved varying the number of trees within a range from 50 to 200 trees, with a step of 5 trees, to determine the optimal model configuration based on accuracy.

The evaluation of a classification model involves a thorough assessment using multiple statistical metrics, each offering insights into different facets of the model’s efficacy (Table 3). The primary measure of the model’s overall success is its accuracy, calculated as the sum of true positives and true negatives divided by the total number of cases. This metric, while fundamental, is complemented by a confusion matrix, which provides a granular view of the model’s predictive errors by categorizing predictions into true positives, true negatives, false positives, and false negatives. The model’s sensitivity, or its ability to accurately identify positive instances, is another critical metric. Conversely, precision measures the accuracy of positive predictions made by the model. The F1 score, which combines precision and sensitivity through their harmonic mean, is crucial in contexts where it is vital to balance the two metrics. The error rate, which calculates the proportion of misclassifications, serves as an additional performance indicator, inversely related to accuracy. Moreover, the model’s discriminative ability is assessed through the ROC curve and its corresponding AUC, quantifying how well the model distinguishes between classes.

By leveraging these metrics, one can derive a multifaceted understanding of a classification model’s performance, encompassing not only its overall accuracy but also its proficiency in identifying and differentiating between classes.

## 3. Results and Discussion

**Figure 4:**
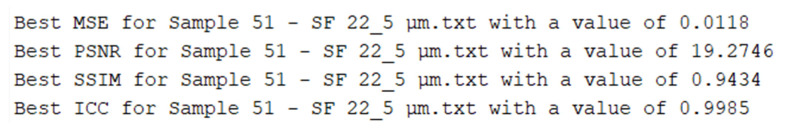
Result of the filtering method and cut-off values selection result for sample 51.

The initial phase of the research involved preparing test samples using a variety of filtering methods: Gaussian regression filter, robust Gaussian regression filter, spline filter, and fast Fourier transform filter. For each of these filtering techniques, cut-off values were analysed at several increments: 2.5 µm, 5 µm, 7.5 µm, 10 µm, 12.5 µm, 15 µm, 17.5 µm, 20 µm, 22.5 µm, and 25 µm. For every instance within the dataset, the optimal filtration method and threshold value were determined based on the performance metrics listed in Table 1, aiming to achieve the highest quality of the resulting image. An example result of selecting the filtering method and cut-off values for sample 51 is shown in Figure 4.

For all samples, the optimal cut-off size was found to be 22.5 µm. Among the selection of filtering methods, only two were identified as yielding the best results: the Gaussian regression filter and the spline filter. Notably, at no point were methods such as the robust Gaussian regression filter or the fast Fourier transform filter chosen. The designed classifier is tasked with selecting the appropriate filtering method, predicted on the assumption that the cut-off value is set at 22.5 µm. When generating data for training the classifier, an index indicative of the optimal model for each instance was recorded in a designated table. In this table, a value of 0 signifies the model employing the Gaussian regression filter method, while a value of 1 denotes the model utilizing the spline filter method. The prepared data were then used to model the classifier.

### Classifier Training Results

**Figure 5:**
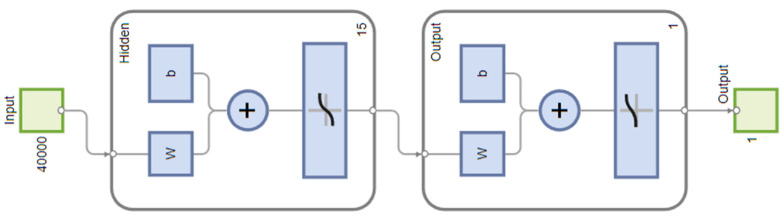
The structure of a neural network.

**Figure 6:**
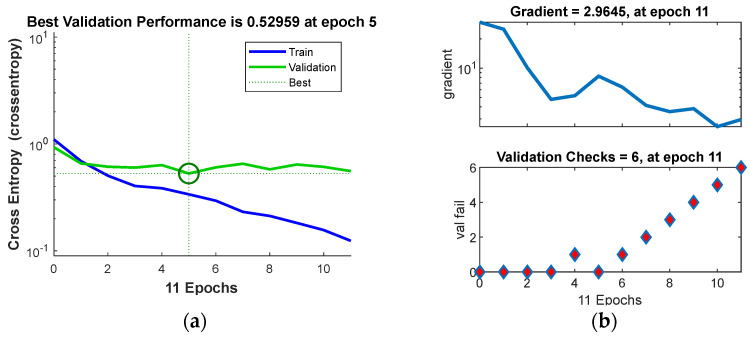
Network training process: (**a**) best validation performance of the predictive model and (**b**) validation checks.

The best neural network modelling results were obtained with 15 neurons in the hidden layer. The learning structure of the neural network is shown in Figure 5. During the classifier training, the optimal model performance was achieved at five epochs, reaching a value of 0.52959, as depicted in Figure 6a.

To prevent neural network overfitting, a validation criterion known as “early stopping” is implemented. The learning process is halted if there is no improvement in the validation set for six consecutive epochs. This means that if the model does not show better performance on the validation set across six learning iterations, the training will be prematurely stopped to avoid the overfitting of the training data. This strategy ensures that the network can generalise its knowledge rather than memorise the specific characteristics of the training set. Figure 6b illustrates the progression of the network’s training process.

**Figure 7:**
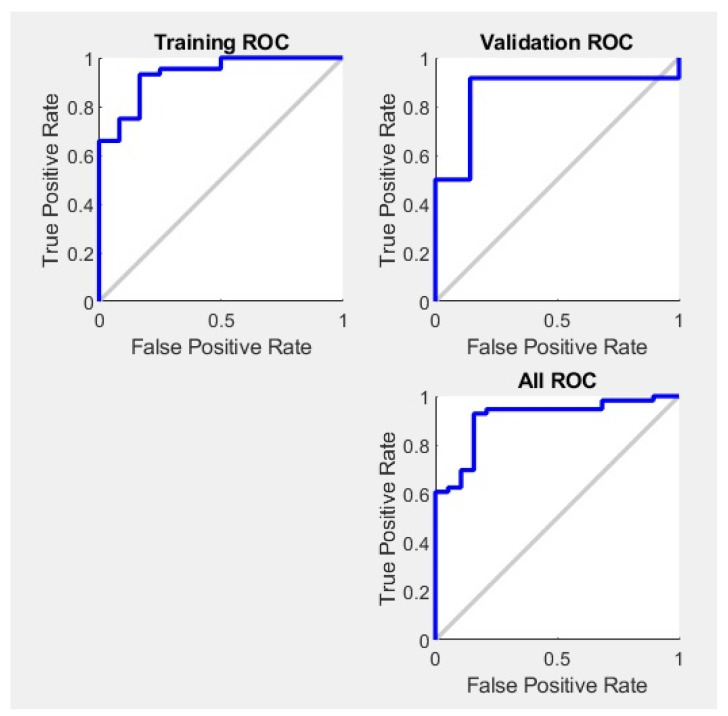
ROC plot for neural network model for datasets.

The effectiveness of a neural network’s fit to data can be evaluated using the ROC curve, which depicts the trade-off between false positives and true positives as the threshold varies from 0 to 1. The closer the curve approaches the top-left corner, the higher the true positive rate achieved with fewer false positives. Ideal classifiers are marked by a curve stretching from the bottom-left corner, through the upper-left, to the upper-right corner of the chart. Figure 7 demonstrates the ROC curve for the neural network model across different datasets, i.e., training, validation, test, and the entire dataset, illustrating this principle. The scalar value represented by an area under the ROC curve (AUC) serves as a quantitative measure of a model’s capacity to distinguish between positive and negative classes over a range of threshold levels. The AUC over all datasets for SVM is 0.89.

**Figure 8:**
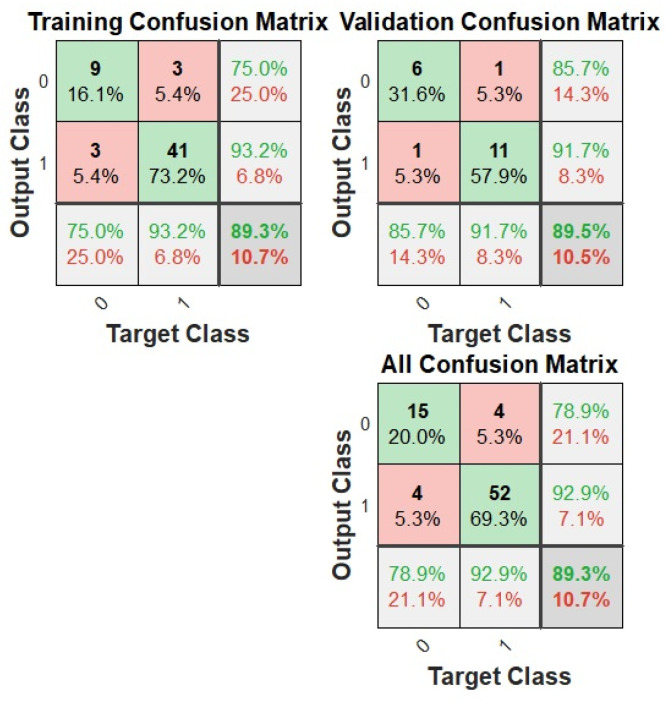
Confusion matrix for neural network model.

Another indicator of the neural network’s effectiveness in fitting the data is the confusion matrix. This matrix showcases the percentages of correct and incorrect classifications. Green squares on its diagonal represent accurate classifications, while red squares indicate errors. The fewer the red squares, the more precise the network is. Figure 8 displays the confusion matrix for training, validation, and test sets, as well as for the entire dataset. The overall accuracy rate achieved was 89.3%.

**Figure 9:**
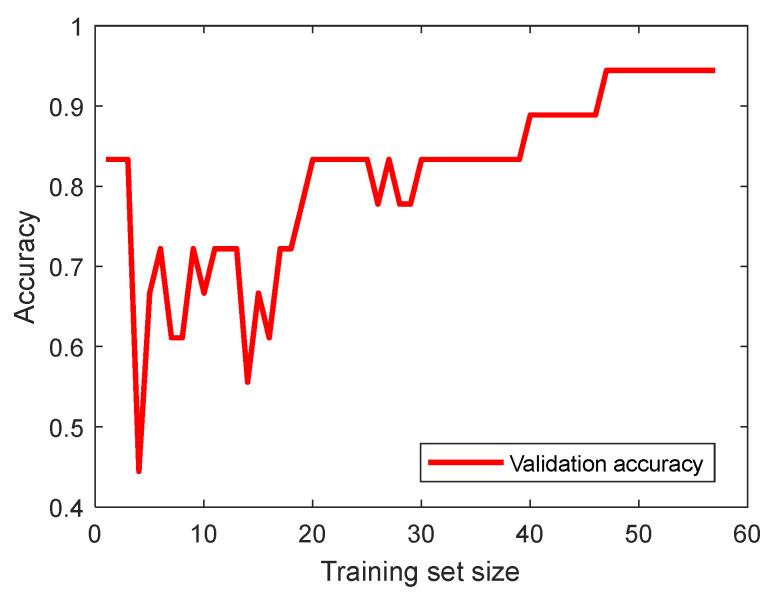
Validation accuracy versus the size of the training set.

Another machine learning method analysed was the support vector machines (SVM). The best SVM modelling results were obtained with the polynomial kernel function. The learning curve, which shows how the accuracy of the validation set changes as the size of the training set increases, is shown in Figure 9.

**Figure 10:**
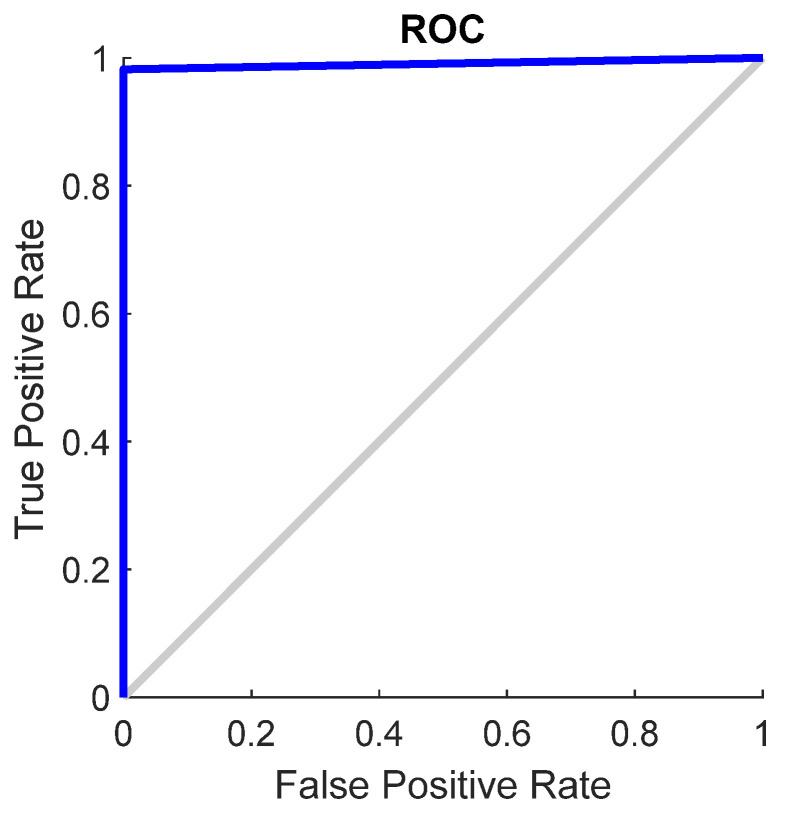
ROC plot for SVM model.

**Figure 11:**
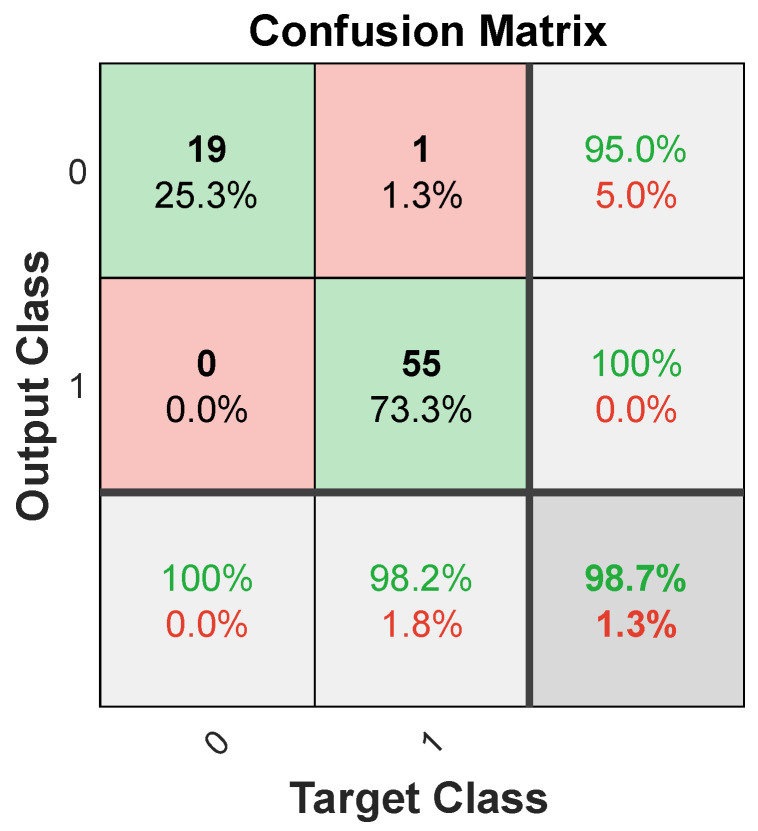
Confusion matrix for SVM model.

Figure 10 shows the ROC curve for the SVM model, and Figure 11 shows the confusion matrix, based on which it can be concluded that the overall accuracy rate achieved was 98.7%. The AUC over all datasets for SVM is 1.00.

**Figure 12:**
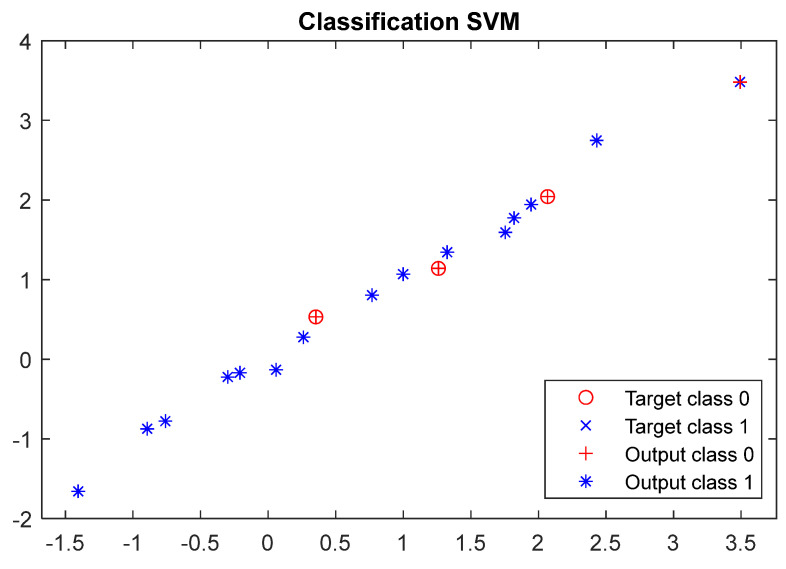
Results of the SVM classifier using test data.

Figure 12 shows the visualisation of the classification results with the SVM model. The resulting scatter plot enables a visual assessment of the SVM model’s performance on the test data. It illustrates the distribution of data points and their grouping according to the actual and predicted classes, highlighting the potential classification errors produced by the model. Data points represented by the different colours signify instances of misclassification by the SVM.

**Figure 13:**
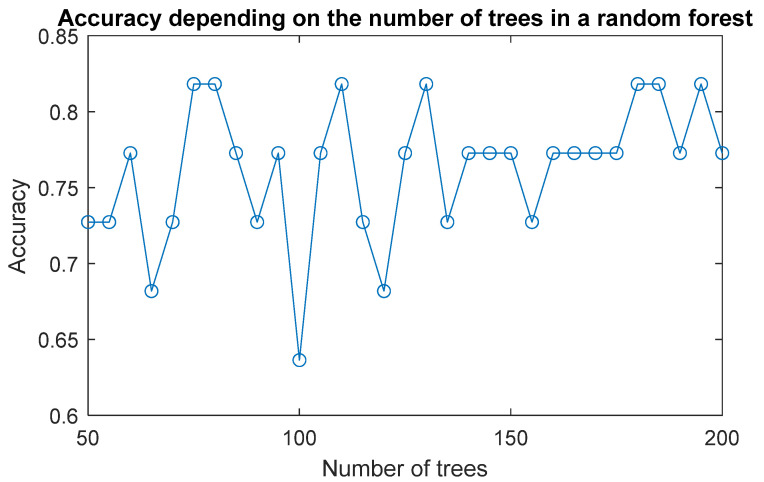
Accuracy depending on the number of trees in a random forest.

The last analysed method for modelling the classifier is decision tree (DT). The process involved varying the number of trees within a range from 50 to 200 trees, with a step of 5 trees. The best DT modelling results were obtained for 75 trees. The graph in Figure 13 shows the accuracy of test data for different numbers of trees in a random forest.

**Figure 14:**
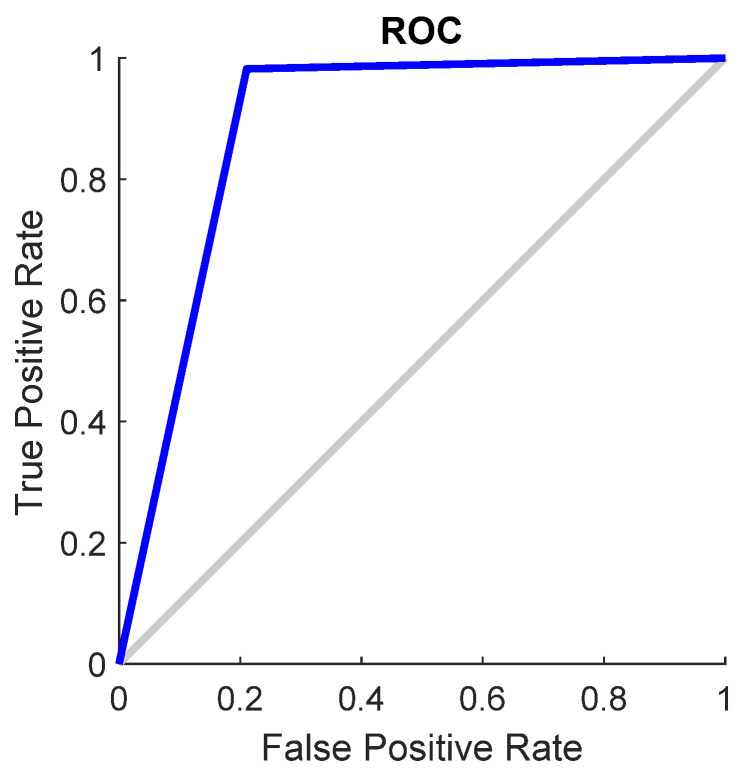
ROC plot for DT model.

**Figure 15:**
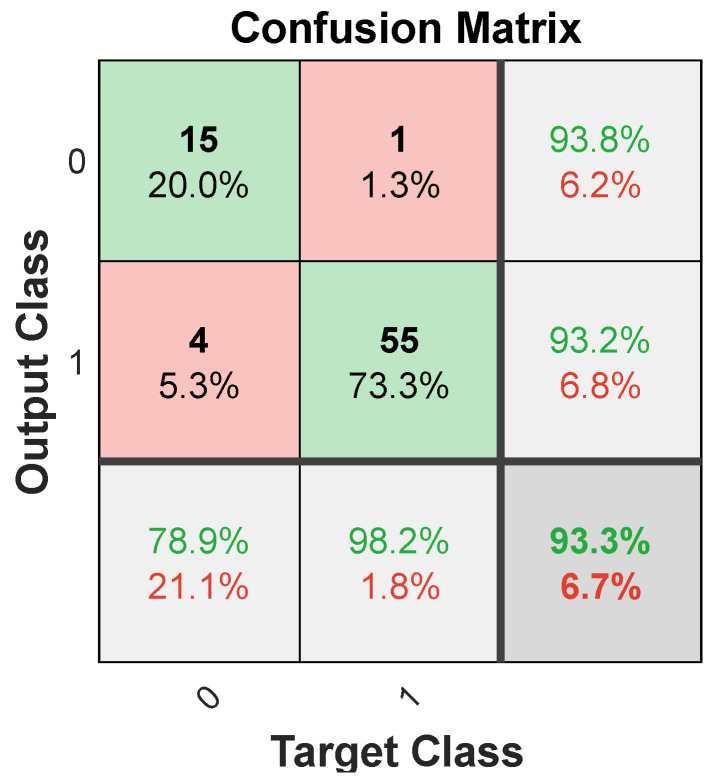
Confusion matrix for DT model.

Figure 14 shows the ROC curve for the DT model, and Figure 15 shows the confusion matrix, based on which it can be concluded that the overall accuracy rate achieved was 93.3%. The AUC over all datasets for DT is 0.82.

The results from the above modelling of the classifier, which is based on measurement data, i.e., roughness measurement, determine the appropriate filtering method using various machine learning techniques. Below, in Table 4, a comparison is made of the performance of each classifier using indicators such as accuracy, sensitivity, precision, F1 Score, and error rate.

Based on Table 4, which presents a comparison of classifiers using different machine learning methods, an analysis of the results and the drawing of conclusions can be conducted as follows. The SVM classifier appears to outperform the other methods with the highest scores for accuracy and sensitivity, achieving 98.67% and 100%, respectively. Such accuracy indicates that the SVM classifier correctly predicts the outcome in the majority of cases. A sensitivity of 100% reflects the SVM’s capability to correctly identify all positive instances, indicating the absence of false negatives in its predictions. The NN classifier exhibits the lowest accuracy among the methods at 89.33%. Its sensitivity, while lower than that of the SVM, remains relatively high at 92.86%, indicating a modest number of false negatives. Precision is shared equally between the SVM and DT classifiers at 98.21%, suggesting a high likelihood of correctly identified positive predictions. The NN classifier also achieves this level of precision, implying that, despite a lower sensitivity, its positive predictions are usually accurate. The F1 score is highest for the SVM at 99.10%, indicating a balanced classifier in terms of precision and sensitivity. The NN and DT classifiers follow with F1 scores of 92.86% and 95.65%, respectively, with the DT showing better performance than the NN. Regarding the error rate, which measures the proportion of incorrect predictions, the SVM classifier exhibits the lowest rate at 1.33%, reinforcing its superior performance. In contrast, the NN and DT classifiers have higher error rates of 10.67% and 6.67%, respectively.

**Figure 16:**
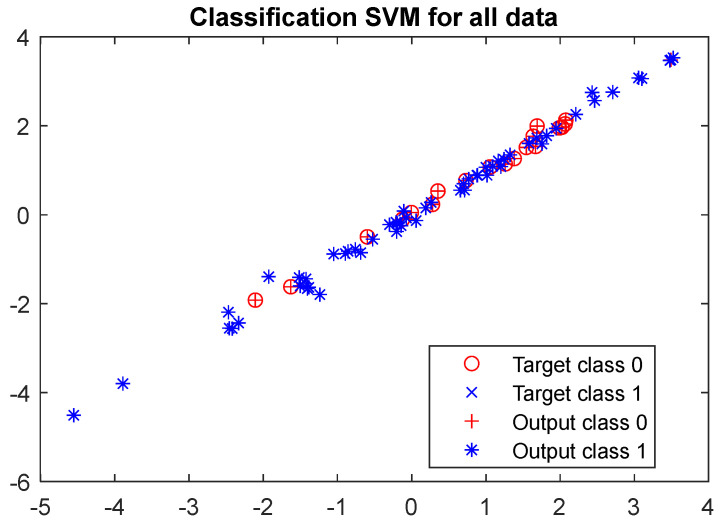
Results of the SVM classifier using all data.

Figure 16 presents a visualisation of the classification outcomes using the SVM model. The scatter plot provided facilitates a visual evaluation of the SVM model’s effectiveness across the entire dataset.

In summary, the SVM classifier is identified as the most effective model according to the presented dataset, offering high accuracy, maximal sensitivity, and a minimal error rate, which positions it as the preferable model, particularly in scenarios where false negatives carry a high cost.

## 4. Conclusions

The presented research focuses on the optimisation of filtering methods to improve surface topography measurements, using machine learning techniques to determine the most effective filtering method and cut-off size based on roughness measurements. The study involves a comprehensive examination of turned surfaces using a white light interferometer for measurement and various digital filters for roughness analysis. The main objective was to select the filter method that minimises measurement distortion using quality indicators such as mean square error (MSE), peak signal-to-noise ratio (PSNR), structural similarity index (SSIM), and image correlation coefficient (ICC).

Three machine learning models were used to predict the optimal filtering method: neural networks (NNs), support vector machines (SVMs), and decision trees (DTs). Each model has its strengths, with NNs being adept at modelling non-linear relationships, SVMs excelling in high-dimensional spaces and achieving precise classification, and DTs providing an intuitive and straightforward classification approach.

The experimental setup involved generating test samples using different filtering methods and cut-offs, with the Gaussian regression filter and the spline filter identified as the most effective methods at a 22.5 µm cut-off. The SVM classifier emerged as the most accurate model, achieving an impressive 98.67% accuracy and 100% sensitivity, demonstrating its superior ability to correctly predict results without false negatives. The SVM’s high precision and F1 score further underline its effectiveness in accurately classifying the filtering methods.

In contrast, the NN model showed the lowest performance of the evaluated methods with an accuracy of 89.33%, while the DT model showed a respectable performance with an accuracy of 93.33%. The SVM model’s low error rate of 1.33% further highlights its reliability and efficiency in this context.

The results of the study highlight the potential of the SVM model as a robust tool for optimising surface measurement and analysis processes, contributing significantly to the accuracy and reliability of surface topography assessments. This research lays the groundwork for further exploration and application of machine learning techniques in surface engineering and metrology.

The use of machine learning algorithms enabled more nuanced and accurate identification of high-frequency measurement errors, outperforming traditional methods that may not capture subtle inaccuracies due to their reliance on less sensitive analytical techniques. The adaptability of machine learning models, including NN, SVM, and DT, to complex and multi-dimensional datasets is critical for dealing with the intricate patterns and variability inherent to surface topography data. This adaptability allows the models to tailor their analytical approaches to the specific characteristics of the data.

This study presents significant implications for various sectors of the industry, particularly those where the precision and quality of surface finishes are crucial. The methodology developed within this research facilitates enhanced precision in the analysis and assessment of surface roughness, a critical aspect of quality control within high-precision manufacturing realms such as aerospace, automotive, tool-making, and microelectronics industries. The optimisation of filtration methods and cut-off sizes based on roughness measurements enables a more effective defect detection and quality improvement of products. Such advancements could lead to the enhancement in production processes by reducing measurement errors and increasing the accuracy of manufactured parts, thereby potentially lowering production costs through the reduction in rejections and corrections. Additionally, the outcomes of this research may contribute towards the development of new measurement devices or software aimed at surface topography analysis. These innovations could feature automatic adjustments of filtration methods to yield the most accurate measurement results, marking a step forward in the capabilities of surface analysis and paving the way for future technological advancements in quality control.

## 5. Limitations and Future Research

The study’s analysis of filtering methods using machine learning models to improve surface topography measurements has some limitations, despite the interesting results obtained. A major limitation is the limited scope of the study in terms of machining conditions, filtering methods, and cut-off values, which may limit the generalisability of the results to other machining processes, materials, or surface conditions. This specificity suggests that the predictive models developed may not perform as well when applied to conditions other than those studied here, thus limiting their broader applicability.

In addition, the limited diversity of the dataset, which consists primarily of surfaces and conditions from a limited set of parameters, could affect the robustness and adaptability of the machine learning models. Expanding the dataset to include a wider variety of materials, machining processes, and environmental conditions could improve the accuracy of the models and their applicability to a wider range of surface measurement challenges.

The complexity of the used machine learning models is also a limitation. While three different models are used in the study, the exploration of more sophisticated algorithms, including deep learning approaches, could provide better insights into the complex relationships between machining parameters and surface quality. However, this exploration could be hampered by the computational intensity required to train more complex models, especially when dealing with larger datasets. This requires considering computational resources and algorithm efficiency.

Another notable limitation is the interpretability of machine learning models. Some models, particularly neural networks, operate as “black boxes”, making it difficult to understand the reasoning behind their predictions. This lack of transparency may be a barrier to the adoption of these models in practice, where understanding the decision-making process is crucial for trust and validation.

Future research could address these limitations by expanding the range of studied machining conditions, incorporating more diverse datasets, and exploring other machine learning models. In addition, investigating the integration of real-time data and the impact of environmental and operational variables could provide a more comprehensive understanding of the factors influencing surface quality. Such advances would not only improve the predictive accuracy and applicability of these models but also contribute to the broader field of surface metrology and manufacturing process optimisation.

## Figures and Tables

**Table 1:** The quality indicators of image reconstruction.

The Quality Indicator	Formula	Explanations of the Symbols
Mean square error (MSE)	$MSE=\sum_{i=1}^{R}\frac{{\left({\hat{y}}_{i}-y_{i}\right)}^{2}}{R}$	R—the total count of pixels in the 2D image *y_i_*—the *i*-th pixel of the pattern image ${\hat{y}}_{i}$—the *i*-th pixel of the reconstructed image *C*_1_ = (0.01 · *L*)^2^, *C*_2_ = (0.03 · *L*)^2^, and *L* is set to 1 when the pixel s is in the range (0, 1) $\mu_{\hat{y}}{,\mu }_{y}$—the local means, $\sigma_{\hat{y}},\sigma_{y}$—standard deviations, $\sigma_{\hat{y}y}$—cross-covariances $\bar{y}$—the average pixel distribution pattern image $\bar{\hat{y}}$—the average pixel distribution reconstruction
Peak Signal-to-noise ratio (PSNR)	$PSNR=10\cdot \log_{10}\left(R^{2}/MSE\right)$	
Structural similarity index (SSIM)	$SIM=\frac{\left(2\mu_{\hat{y}}\mu_{y}+C_{1}\right)\left(2\sigma_{\hat{y}y}+C_{2}\right)}{\left(\mu_{\hat{y}}^{2}+\mu_{y}^{2}+C_{1}\right)\left(\sigma_{\hat{y}}^{2}+\sigma_{y}^{2}+C_{2}\right)}$	
Image correlation coefficient (ICC)	$ICC=\frac{\sum_{i=1}^{R}\left(y_{i}-\bar{y}\right)\left({\hat{y}}_{i}-\bar{\hat{y}}\right)}{\sqrt{\sum_{i=1}^{R}{\left(y_{i}-\bar{y}\right)}^{2}\sum_{i=1}^{R}{\left({\hat{y}}_{i}-\bar{\hat{y}}\right)}^{2}}}$	

**Table 2:** Model description.

Model Index	Filtration Method
1	Gaussian regression filter
2	Robust Gaussian regression filter
3	Spline filter
4	Fast Fourier transform filter

**Table 3:** The quality indicators of classifier selection.

Model Index	Formula	Explanations of the Symbols
Accuracy	$Accuracy\;=\;\frac{TP\;+\;TN}{TP\;+\;TN\;+\;FP\;+\;FN}$	*TP*—true positives *TN*—true negatives *FP*—false positives *FN*—false negatives
Sensitivity	$Sensitivity\;=\;\frac{TP}{TP\;+\;FN}$	
Precision	$Precision\;=\;\frac{TP}{TP\;+\;FP}$	
F1 score	$F1\;Score\;=\;2\frac{Precision\;*\;Sensitivity}{Precision\;+\;Sensitivity}$	
Error rate	Error Rate = 1 − Accuracy	

**Table 4:** Comparison of classifiers obtained using different machine learning methods.

Quality Indicators	NN	SVM	DT
Accuracy [%]	89.33	98.67	93.33
Sensitivity [%]	92.86	100.00	93.22
Precision [%]	92.86	98.21	98.21
F1 Score [%]	92.86	99.10	95.65
Error Rate [%]	10.67	1.33	6.67

## Data Availability

Data supporting this study are included within the article.
